# From Mechanisms to Management: Tackling In-Stent Restenosis in the Drug-Eluting Stent Era

**DOI:** 10.1007/s11886-025-02193-z

**Published:** 2025-02-11

**Authors:** Luigi Spadafora, Rossella Quarta, Giovanni Martino, Letizia Romano, Francesco Greco, Antonio Curcio, Tommaso Gori, Carmen Spaccarotella, Ciro Indolfi, Alberto Polimeni

**Affiliations:** 1 Department of Medical-Surgical Sciences and Biotechnologies, Sapienza University of Rome, Latina, Italy; 2 Department of Pharmacy, Health and Nutritional Sciences, University of Calabria, 87036 Rende, Italy; 3 Division of Cardiology, Department of Medical and Surgical Sciences, Magna Graecia University, 88100 Catanzaro, Italy; 4 Division of Cardiology, Annunziata Hospital, 87100 Cosenza, Italy; 5 Division of Interventional Cardiology, Annunziata Hospital, 87100 Cosenza, Italy; 6 Zentrum Für Kardiologie, Kardiologie I, University Medical Center Mainz and DZHK Standort Rhein-Main, Mainz, Germany; 7 Division of Cardiology, Department of Advanced Biomedical Sciences, University of Naples Federico II, 80126 Naples, Italy

**Keywords:** Percutaneous coronary interventions, coronary stent, in-stent restenosis, DES

## Abstract

**Purpose of review:**

Drug-eluting stent (DES) technology has greatly enhanced the safety and effectiveness of percutaneous coronary interventions (PCI). The aim of the present paper is to provide a comprehensive review of in-stent restenosis (ISR), focusing on the contemporary DES era, including its incidence, mechanisms, and imaging characterization.

**Recent findings:**

Despite the widespread use of DES and numerous improvements, recent clinical data indicate that ISR still occurs in 5–10% of PCI procedures, posing a considerable public health issue.

**Summary:**

The incidence, morphology, and clinical implications of ISR are determined by a complex interplay of several factors: the patient, stent, procedure, and vessel and lesion-related factors. Advancements in intracoronary imaging have provided greater insight into its patterns and underlying causes. Over time, treatment strategies have evolved, and current guidelines recommend an individualized approach using intracoronary imaging to characterize ISR’s underlying substrate.

## Introduction

Drug-eluting stents (DESs) were introduced as a solution to the high incidence of in-stent restenosis (ISR) that had been observed with the use of bare-metal stents (BMSs) [1]. These stents modify the healing process after deployment, reducing neointimal hyperplasia (NIH) and ISR rates ranging from 5 to 10% [2, 3]. Despite advancements regarding the stent platform, antiproliferative agents, and polymers, the incidence of ISR remains similar with new generation DESs, creating a therapeutic issue [4, 5]. ISR is defined as luminal narrowing exceeding 50% of the stented coronary segment or within 5 mm of a stent edge [3, 6]. The mechanisms behind restenosis include multiple factors, and intracoronary imaging methods can often highlight these mechanisms [7]. In this review, we examine the incidence, mechanisms, assessment, and treatment options for patients with DES-ISR.

## Definition of ISR

ISR is usually defined at angiography in binary terms as a > 50% diameter stenosis within 5 mm of the stent edges [1, 4]. While this pragmatic definition is easily accessible and has an acceptable correlation with physiological measures, it is intrinsically dependent on the assumption of cross-sectional circularity of NIH and on the capacity to obtain the most informative (minimal diameter) projection [8]. On the other hand, intravascular imaging provides a comprehensive cross-sectional evaluation of the artery, enabling direct visualization and measurement of the stent, neointimal, and luminal areas [9].

## Epidemiology and Clinical Presentation

Since DESs have proven to be more effective than BMSs over time, BMSs have disappeared from routine clinical practice [10]. Improvements in stent platforms, polymers, and antiproliferative drugs have led to superior performance of second-generation DESs compared to the earlier versions [11]. In the United States, 10% of percutaneous coronary interventions were performed to treat ISR [2]. The timing of DES-ISR presentation has been delayed, now occurring several years after stent deployment [12]. Although DES technology has seen recent advancements, the persistently stable rate of ISR observed in clinical practice may be attributed to the gradual accumulation of ISR over time, late neoatherosclerosis, and delayed healing [13]. The management of DES-ISR is deemed more complex and results in poor clinical outcomes as compared to revascularization of new lesions and it was previously considered a pathological condition that progressed slowly and was generally benign [14]. ISR, which can frequently manifest as an ACS, is now more widely acknowledged to be malignant [1, 14]. High-sensitivity troponin levels are frequently elevated in individuals who have been diagnosed with ISR in clinical practice, sometimes meeting the criteria for myocardial infarction (MI) [15]. A study found that elevated lipoprotein (a) [Lp(a)] levels are linked to a higher risk of major adverse cardiac events (MACE) in patients undergoing percutaneous coronary intervention for in-stent restenosis, underscoring the importance of considering Lp(a) levels in patient risk assessment [16]. Intravascular imaging has shown acute plaque rupture and stent thrombosis (ST) in DES-ISR patients, demonstrating that these diseases comprise a portion of ISR presentations [17]. These diseases, previously thought to be separate clinical entities, may be connected to ISR by neoatherosclerosis [18]. Notably, ACS presentation has also been linked to an increased incidence of angiographic restenosis and recurrent major adverse cardiovascular events (MACE) [19]. In addition, in their study published in the American Journal of Cardiology, Zhang et al. found that recurrent ISR patients face significantly higher risks of adverse cardiovascular outcomes compared to first-time ISR patients [20].

## Basic Mechanisms and Pathophysiology

Many traditional pathogenic factors involved in BMS-ISR, including clinical, biological, mechanical, and procedural issues, are also relevant to DES-ISR (Table 1) [4]. The molecular mechanism underlying the formation of the neointima has been elucidated and involves the activations of the MAPKKs [21]. While DESs reduce the growth of neointima compared to BMSs, hypersensitivity to the polymer and drug, local inflammation, and delayed healing are the primary contributors to NIH of DES-ISR [22]. Clinical presentation, etiology, lesion form, and responsiveness to treatments vary significantly between different stent technologies [23]. Vascular smooth muscle cells (VSMCs) and extracellular matrix are distributed throughout the neointimal tissue in BMS-ISR. This condition achieves its peak around 6 months after deployment [24]. The coating on DESs, on the other hand, prevents and triggers delayed NIH [25]. In contrast to the diffuse pattern present in BMS-ISR, DESs are linked to a more focal pattern and frequently involve the stent margins [1]. Moreover, studies using optical coherence tomography (OCT) have shed light on the distinction between early ISR, which is characterized by uniform NIH, as compared to late ISR which is characterized by neoatherosclerosis with thin-cap fibroatheroma and lipid-rich neointima[26]. Neoatherosclerosis refers to the formation of an atherosclerotic lesion within the neointima of a previously stented arterial segment [6, 27]. The pathologic features of neoatherosclerosis might range from intimal thickening with lipid infiltration to ruptured thin-cap fibroatheroma with thrombosis [27]. Compared to BMSs, first-generation DESs show a higher prevalence of diffuse in-stent thin-cap neoatheroma and neoatherosclerosis, potentially playing a role in the incidence of late stent failure linked to DESs [28]. OCT is the recommended imaging technique to identify neoatherosclerosis given that intravascular ultrasonography holds several drawbacks for the assessment of neoatherosclerosis [29, 30]. With regard to coronary physiology, there's limited evidence on its role in ISR management [3, 8]. Although studies indicate conservative treatment of moderate ISR (40–70%) with an FFR ≥ 0.75 is safe, no randomized trials support an FFR-guided ISR treatment strategy [3, 8].

**Table 1:** In-stent restenosis (ISR) mechanisms

Patient-related	Stent and Procedural-related	Vessel- and Lesion-related
Drug Resistance	Stent underexpansion	Small vessels
Hypersensitivity	Stent Fracture	Calcified lesions
-HypersensitivitytoStent Platform	Stent thickness	Bifurcation lesions
- Hypersensitivity to Drug	Stent gap	Ostial lesions
- Hypersensitivity to Polymer	Overlapping stents	
Clinical Factors	Non-uniform drug distribution	
- Diabetes	Geographical lesion mass or barotraumaoutsidestented segment	
- Chronic kidney disease		
- Advanced age		
- Female sex		
- Abnormal body mass index		

## Patient-related Factors:

### Drug Resistance

Cytostatic effects are produced by sirolimus and its analogues, which inhibit the mammalian target of rapamycin's activity by terminating the cell cycle in the G1 phase, which decreases VSMCs migration and proliferation [31]. The primary action of paclitaxel, which has a cytotoxic impact and binds particularly to the beta-tubulin subunit of microtubules, involves disrupting the dynamics of these structures and preventing them from depolymerizing [32]. Moreover, genetic changes can affect a drug's sensitivity, imparting resistance to drugs of the -limus family or paclitaxel [33].

## Hypersensitivity

Given that DESs consist of three components—the stent platform, antiproliferative drug, and polymer—any of these elements has the potential to provoke an allergic reaction.

### Hypersensitivity to Stent Platform

The most common stent platform material for BMS and first-generation DES is stainless steel (316L SS), which contains nickel, chromate, and molybdenum [34]. These materials have been evidenced to be a potential trigger for ISR [35]. In contrast, the platform material in newer generation DESs is cobalt chromium, which contains less nickel than 316L stainless steel and appears less likely to induce hypersensitivity or an adverse proliferative response [22].

### Hypersensitivity to Antiproliferative Drugs

First-generation DESs primarily use sirolimus and paclitaxel agents as drugs [34]. Numerous studies, including histological studies on animals, have indicated that these drugs can trigger allergic reactions [36]. In contrast, second-generation DESs use Everolimus and Zotarolimus as carrier drugs that, compared with sirolimus, can be used at lower drug concentrations and have reduced toxicity [37].

### Hypersensitivity to Polymers

Durable polymers that persist on the stent surface after drug elution raised questions about their potential role in allergic reactions [38]. First-generation DES polymers are strongly associated with an inflammatory response that persists beyond 180 days for up to 2 years [39]. This contrasts with second-generation DESs showing a reduced inflammatory reaction, lasting between 90 days and 12 months [40].

## Clinical Factors

Patient demographics associated with ISR have also been extensively investigated. Diabetes mellitus, chronic kidney disease, advanced age, female sex, and abnormal body mass index are clinical predictors of ISR [12, 41]. Although early reports were inconsistent in linking diabetes and ISR, now according to multiple studies diabetic patients suffer worse outcomes after DES implantation [42–45].

## Stent and Procedural-related Factors:

### Stent Underexpansion

While acute stent underexpansion is commonly caused by stent recoil, chronic stent underexpansion is mostly characterized by calcified plaque [46]. This phenomenon is easily detected by IVUS and OCT and according to several studies, stent underexpansion is a powerful predictor of ISR after DES implantation [47]. One of the reasons is that blood flow is disturbed by the stent underexpansion, leading to high shear stress and creates a flow reversal area [48]. High shear stress alters leukocyte and platelet adherence, endothelial gene expression, cytoskeletal architecture, and cytokine and growth factor accumulation, predisposing the region to neointimal development [49].

## Stent Fracture

A stent fracture refers to the complete or partial breakage of a stent that was initially continuous following its implantation [50]. Stent fractures may contribute to ISR as a result of localized trauma caused by movement at the stent edges and the surrounding vessel [51]. Additionally, a stent fracture negatively affects local drug delivery and removes the metal scaffolding support at the targeted spot [52]. A stent fracture may be termed either partial or total depending on several factors[53]. Key factors that predict stent fracture include the location in the saphenous vein graft, the length of the stent, and placement in the right coronary artery [54].

## Stent Thickness

Thicker stent struts have been associated with an augmented ISR risk, especially in small vessels [55]. ISR is related to strut thickness, form, and placement at intersections between struts [56]. Stents designed to optimize hemodynamics are associated with consistent endothelial shear stress (ESS) across the inner surface, leading to a reduced risk of ISR, irrespective of stent type [56, 57]. Lastly, intracoronary imaging studies have shown that thinner stent struts are associated with improved local blood flow dynamics and a reduced incidence of ISR [58]. Similar results arose from more extensive RCTs comparing thinner-strut DESs with thicker-strut DESs [58].

## Overlapping Stents

While some researchers have suggested that the safety and effectiveness of overlapping DESs are comparable to using a single long stent or multiple short ones, more recent studies have raised concerns about potential adverse outcomes [59]. Stent overlap has been linked, regardless of the type of stent, to increased ISR and lumen loss because of late healing and higher inflammation [60].

## Non-uniform Drug Distribution

Homogeneity of drug elution may be hampered by local blood flow changes, strut overlap, and polymer deterioration, according to physiological and computational models [61]. Variations in drug release, including coating degradation or uneven stent expansion, can lead to areas within the stented segment with inadequate drug distribution, increasing the likelihood of ISR [62]. These variations are reflective of the metal-to-artery ratio of the various types of DES stent platforms [63].

## Vessel- and Lesion-related

### Small Vessels

ISR frequently occurs after stent placement in small vessels, often due to factors such as reduced luminal area post-procedure, increased vessel damage and recoil, and higher metallic density [1, 55, 64]. The overstretch theory proposes that excessive vessel stretching during stent placement may lead to adverse outcomes [65]. Some studies suggest a possible adverse effect of overstretching on NIH, while others suggest no significant effect or even potential benefit [1, 65]. Beneficial effects are attributed to a larger balloon-to-artery ratio, commonly referred to as the 'bigger is better' principle [39]. This approach involves using a larger balloon to achieve appropriate apposition of the stent to the vessel wall, which may result in better outcomes, as it results in several studies [66].

## Calcified Lesions

Coronary artery calcification (CAC) independently predicts major cardiovascular incidents and can complicate PCI due to issues like inadequate stent expansion and increased risks of in-stent restenosis and thrombosis [67, 68]. The primary cause of CAC is vascular smooth muscle dysfunction, which triggers a process leading to calcium buildup in the vessel walls [67, 68]. Computed tomography coronary angiography (CCTA) effectively identifies coronary vessel calcification non-invasively, and a study found that the calcium score is strongly associated with an increased risk of ISR in patients with coronary stents [69]. Hemodialysis patients are at higher risk of ISR following DES placement due to a higher presence of calcified nodules (CN) in their coronary arteries [46]. Histopathological studies confirm CN as a common cause of ISR in hemodialysis patients, with poorer outcomes observed in ISR lesions with CN [46, 70]. Recurrence after CN treatment is frequent, suggesting a complex relationship between CN and ISR [46, 71–73].

## Bifurcation Lesions

ISR is a potential complication following stent placement in bifurcation lesions [74, 75]. Multiple factors play a role in the development of ISR in bifurcation lesions, such as stent underexpansion, malapposition, and insufficient coverage of the side branch at the bifurcation [76]. These factors can lead to areas of turbulent blood flow, promoting the development of neointimal hyperplasia (NIH) and ISR[1].

## Ostial Lesions

Ostial lesions are linked to a heightened risk of ISR following stent implantation [77]. These lesions are found at the origin of a major coronary artery, where the vessel wall is thinner and the lumen is broader compared to distal segments [78]. As a result, stent placement in this region can be challenging and may lead to incomplete stent apposition, malapposition, or under-expansion, which are all risk factors for ISR [78]. The presence of calcified plaques, thrombus, and lipid-rich plaques can also contribute to ISR in ostial lesions [1]. A recent study focused on right coronary artery (RCA) ostial lesions, employing IVUS to analyze 139 ISR lesions [79]. The primary ISR mechanisms identified included neointimal hyperplasia, neoatherosclerosis, stent-related issues like underexpansion, fracture, or deformation, and protruding calcified nodules [79].

## Angiographic Classification of ISR

While the Mehran classification is historically relevant for ISR in bare-metal stents (BMS), the Waksman classification has been developed to address the distinct challenges associated with drug-eluting stents (DES) by identifying underlying mechanical and biological factors of in-stent restenosis.

## Mehran ISR Classification

While ISR can be detected through angiography, intracoronary imaging offers greater sensitivity, providing insight into the underlying causes of ISR. The BMS-ISR-specific angiographic categorization described by Mehran et al. established 4 types of ISR [80] (Table 2). The prognostic predictors of repeat revascularization for BMSs on which this classification scheme is based are less relevant to DESs. These historical criteria do not reveal the mechanism of stent failure or the appropriate treatment course, making them less applicable to DESs.

**Table 2:** Mehran's angiographic classification of in-stent restenosis (ISR)

Type of ISR	Characteristics	Occurrence
I—focal	Length < 10 mm	42%
-IA	The articulation or gap between stents	
-IB	The proximal or distal margin	
-IC	The body of the stent	
-ID	Multifocal	
II—diffuse	Length > 10 mm, not exceeding the edges of the stent	21%
III—proliferative	Exceeding the edges of the stent	30%
IV—occlusive	Total occlusion with TIMI 0	7%

## Imaging-based Classification of ISR

### Waksman ISR Classification

A classification system has been proposed that categorizes ISR based on the specific mechanisms driving its development (Table 3) [5]. The Waksman ISR Classification categorizes the causes into mechanical (Type I), biological (Type II), and mixed (Type III), as well as including chronic complete occlusions (Type IV) and DES-ISR lesions previously treated with more than two stents (Type V) [5]. This classification is critical to appropriately categorize and individualize DES-ISR treatment [6]. In brief, the priority of the treatment in which ISR appears to be caused by stent undersizing or underexpansion (Waksman type I A) is the expansion of the original stent to a minimum stent area of at least 4.5mm^2 ^[5, 6]. The identification of the nature of the ISR tissue can guide the choice between high-pressure dilation with non-compliant balloons (fibrous or lipidic neointima / plaque), debulking devices (rotational or orbital atherectomy), litotripsy, or scoring/cutting devices (calcific neointima) (Waksman type II C) [5, 6]. Of note, the use of these devices is considered off-label in several countries due to a potential hazard of entrapment. Furthermore, imaging allows discriminating stent underexpansion (lack of expansion of the correctly sized stent during implantation) from undersizing (choice of a small stent), the former being mostly due to calcific lesions that require off-label intravascular lithotripsy [81–88]. Regarding the choice of device for treating ISR, several clinical trials have provided somewhat conflicting results but generally demonstrated the comparable efficacy of DES and drug-eluting balloons (DEBs) [18, 89–95]. In the retrospective study by Tada et al., imaging-based morphological assessment of the ISR tissue by OCT was used to guide the selection of the appropriate treatment strategy [96]. In the absence of a comparator group without imaging, the authors confirmed the superiority of DEB and DES as compared to high-pressure PTCA with standard balloons [96]. In the especially difficult case of resistant or recurrent ISR (Waksman type V), intracoronary imaging offers high spatial resolution, allowing for precise identification of the number of stent layers within each lesion segment and assessing focal expansion [5, 6]. In this setting, imaging identifies in detail which segments should not be treated with additional stent layers but rather with a DEB [5].

**Table 3:** Waksman classification of in-stent restenosis (ISR)

Type	Definition	Treatment options
I	Mechanical	
	- Underexpansion (IA) - Stent Fracture (IB)	- High-pressure balloon - DES
II	Biologic	
	- Intimal hyperplasia (IIA) - Neoatherosclerosis, noncalcified (IIB) - Neoatherosclerosis, calcified (IIC)	- Balloon, DCB, DES, and VBT - DCB and DES - Scoring balloon, ELCA, and RA
III	Mixed pattern: Combined mechanical and biologic etiology	Scoring balloon, ELCA, and RA High-pressure balloon with DCB, DES, or VBT
IV	Chronic total occlusion	DCB or DES, VBT for multiple layers, CABG as needed
V	> 2 layers of stent	Balloon, DCB, VBT, and CABG

CABG—coronary artery bypass graft; DCB—drug-coated balloon; DES—drug-eluting stent; ELCA—excimer laser coronary atherectomy; RA—rotational atherectomy; VBT—vascular brachytherapy

## Comparison of the Two Classifications

In summary, the Waksman classification allows for a treatment algorithm that matches the ISR etiology with appropriate therapeutic options, which is particularly valuable in cases of DES-ISR. This approach contrasts with the Mehran classification, which, while effective for BMS cases, provides limited guidance in the management of complex ISR cases in the DES era. Therefore, clinicians may consider using the Waksman classification when intracoronary imaging reveals detailed ISR mechanisms, enhancing the decision-making process for targeted interventions (Table 4).

**Table 4:** Comparison of the two classifications

**Classification**	**Mehran**	**Waksman**
**Applicability**	Primarily BMS-ISR	Primarily DES-ISR
**Categories**	Focal, Diffuse, Occlusive	Mechanical, Biological, Mixed
**Clinical Utility**	Rehospitalization prediction	Guides tailored ISR treatment
**Intervention Guidance**	Minimal	High, specifies techniques per ISR type
**Pathology-Based**	No	Yes

## Imaging-based Patterns of ISR

**Fig. 1:**
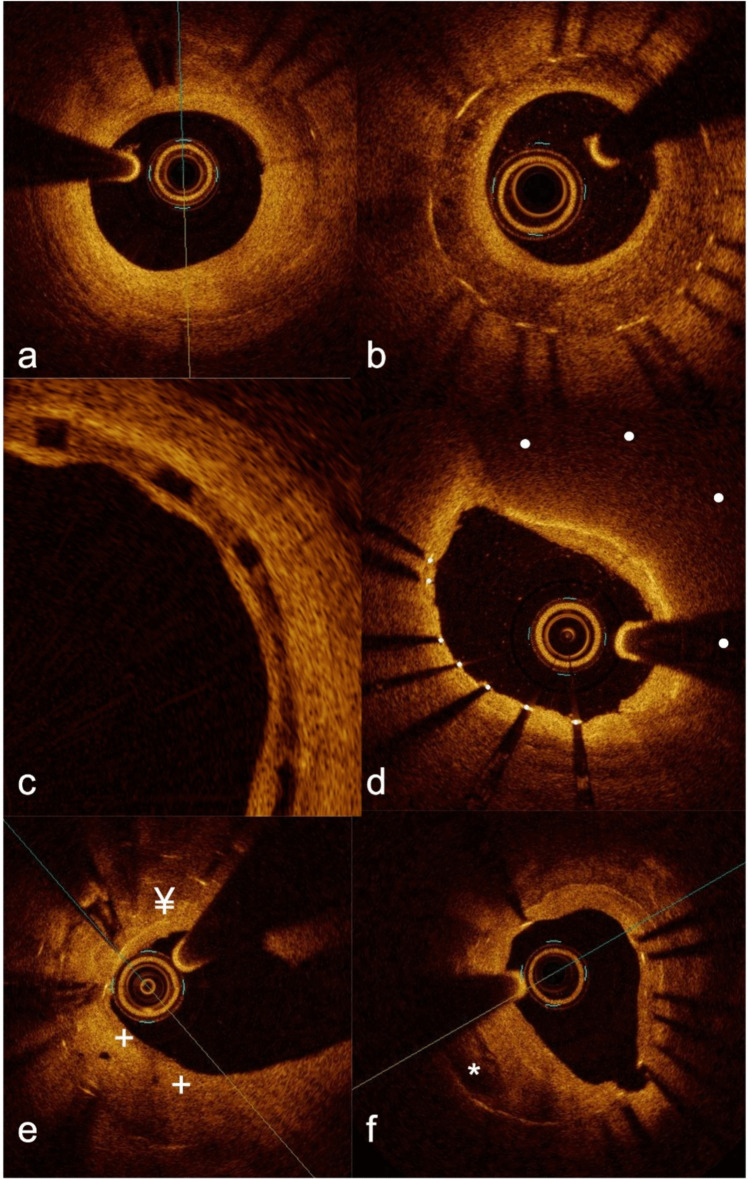
Multiple patterns of in-stent restenosis. As compared to IVUS, OCT offers higher resolution and detail. **a** High-backscattering, low-attenuation tissue compatible with fibrotic neointima. **b** Heterogeneous, layered neointima with areas of different backscattering pattern. **c**, Peri-strut low-intensity areas. **d**, in-stent lesion with high attenuation and low backscattering, typical of atheromas. The position of the (invisible here) stent struts is marked by the *white dots*. **e**, High backscattering, high attenuation spots compatible with macrophage pools or microcalcifications (¥) and microvessels (+). **f**, Calcific lesion within the neointima. Calcific nodule with high superficial backscattering and high distal attenuation is marked by the *asterisk*

Beyond the quantification of ISR, imaging provides morphological information and a potential insight on the mechanism of NIH [1, 3]. Based on its backscattering and attenuation properties, neointimal tissue can be classified in multiple different patterns [97]. In summary, this pattern, characterized by a uniform high backscattering on OCT or low backscattering on IVUS without significant attenuation (Fig. 1a), is typical of bare metal stent restenosis and indicates stable, slow-growing fibrous tissue [97]. This type of restenosis was present in 45% of the ISR lesions in a recent study [98]. In contrast, the deposition of fibrin, platelets, or extracellular matrix proteoglycans that might be associated with inflammatory reactions determine a heterogeneous or layered neointima aspect, with multiple focal backscattering patterns (Fig. 1b) [97]. Compatible with this, inhomogeneous neointima is more frequent in DES than BMS, along with a more focal angiographic pattern [99]. Peri-strut low-intensity areas also are typical findings associated with the deposition of fibrin and proteoglycans [100] (Fig. 1c). Eventually, the development of neoatherosclerosis (i.e., infiltration of plaques from outside the stent or de-novo atherosclerosis within the neointima) represents an increasingly common cause of stent failure [101]. Considering new plaques, imaging may identify lipid-laden neointima (low-backscattering areas with poorly defined margins and high attenuation on OCT or low attenuation on IVUS, Fig. 1d), intimal disruption and thrombus [102].

## Identification of the Mechanical Cause of ISR

### IVUS and OCT

**Fig. 2:**
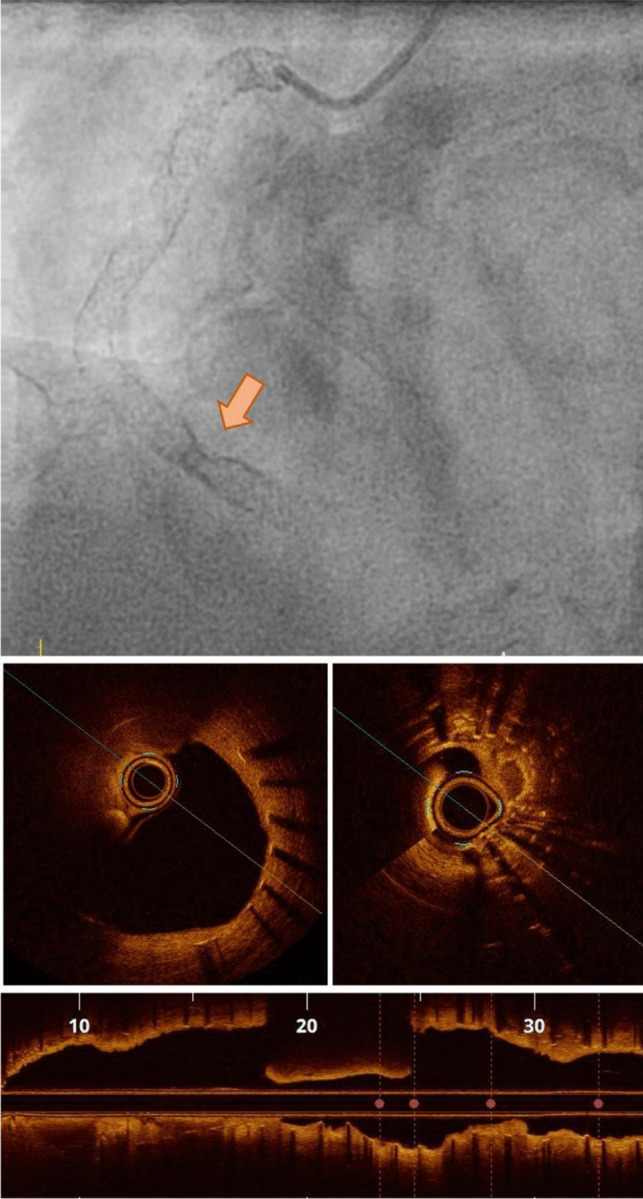
Severe expansion and in-stent restenosis (*red arrow*)

**Fig. 3:**
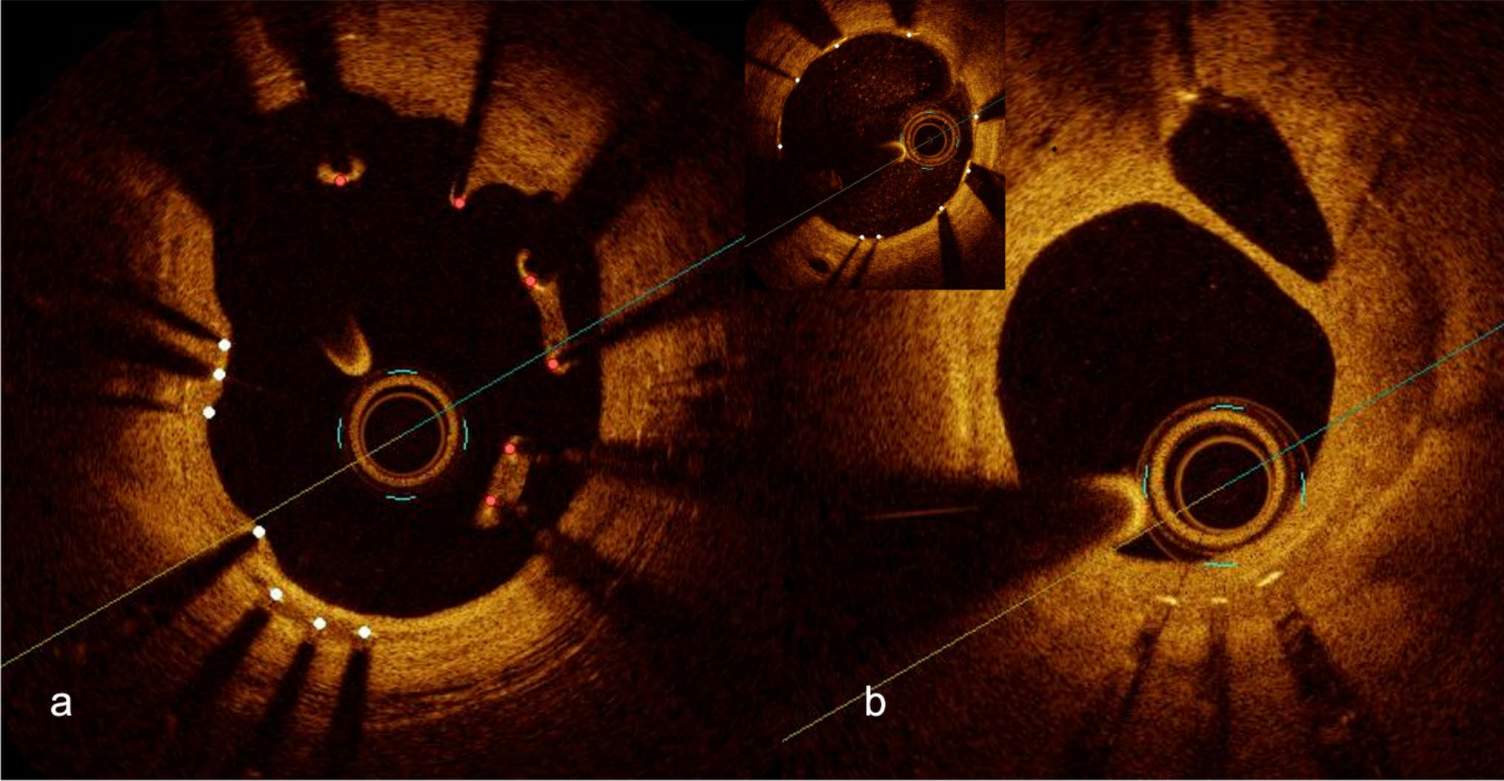
**a** Malapposition 6 months after implantation. The stent struts are marked by *white dots* (*red:* malapposed struts). **b **Symmetric vs inhomogeneous distribution of the stent struts. **b** also shows a double lumen, likely due to formation of a fibrin bridge during stent healing.

**Fig. 4:**
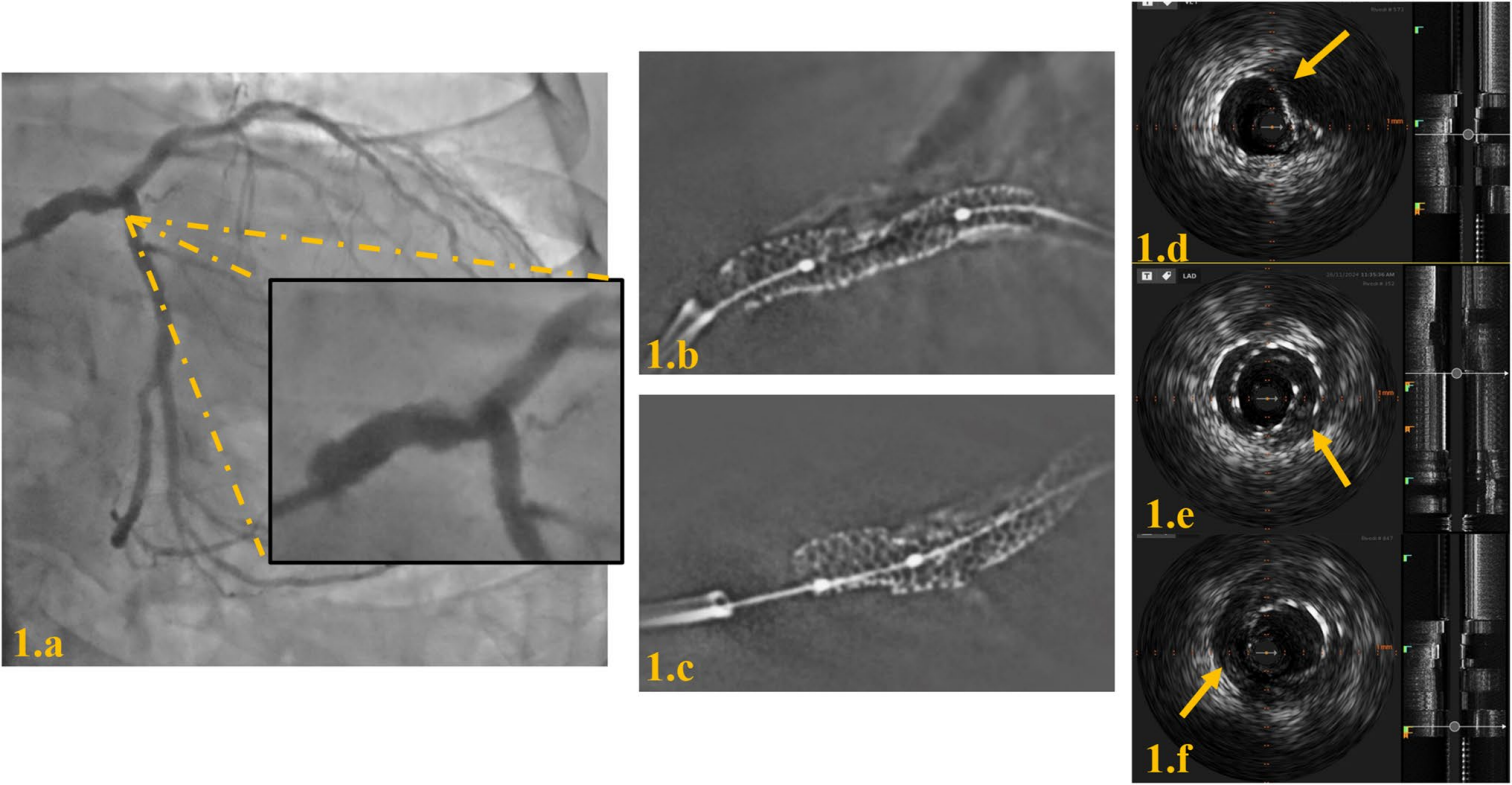
Severe stent underexpansion and malapposition in a calcified coronary lesion. 1.a: Coronary angiography reveals a heavily calcified lesion with suboptimal stent deployment, evidenced by vessel narrowing and incomplete stent expansion. 1.b: Stent enhancement imaging highlights zones of inadequate stent apposition and expansion. 1.c: Magnified imaging demonstrates discontinuity in the stent struts, indicative of a probable stent fracture. 1.d: IVUS cross-sectional view shows severe calcification (shadow cone effect) and marked stent underexpansion. 1.e: IVUS cross-sectional view illustrates classical features of stent fracture, including strut discontinuity, malapposed segments, and overlapping stent structures.1.f: IVUS cross-sectional view underscores stent malapposition, with the stent diameter clearly undersized relative to the vessel lumen

**Fig. 5:**
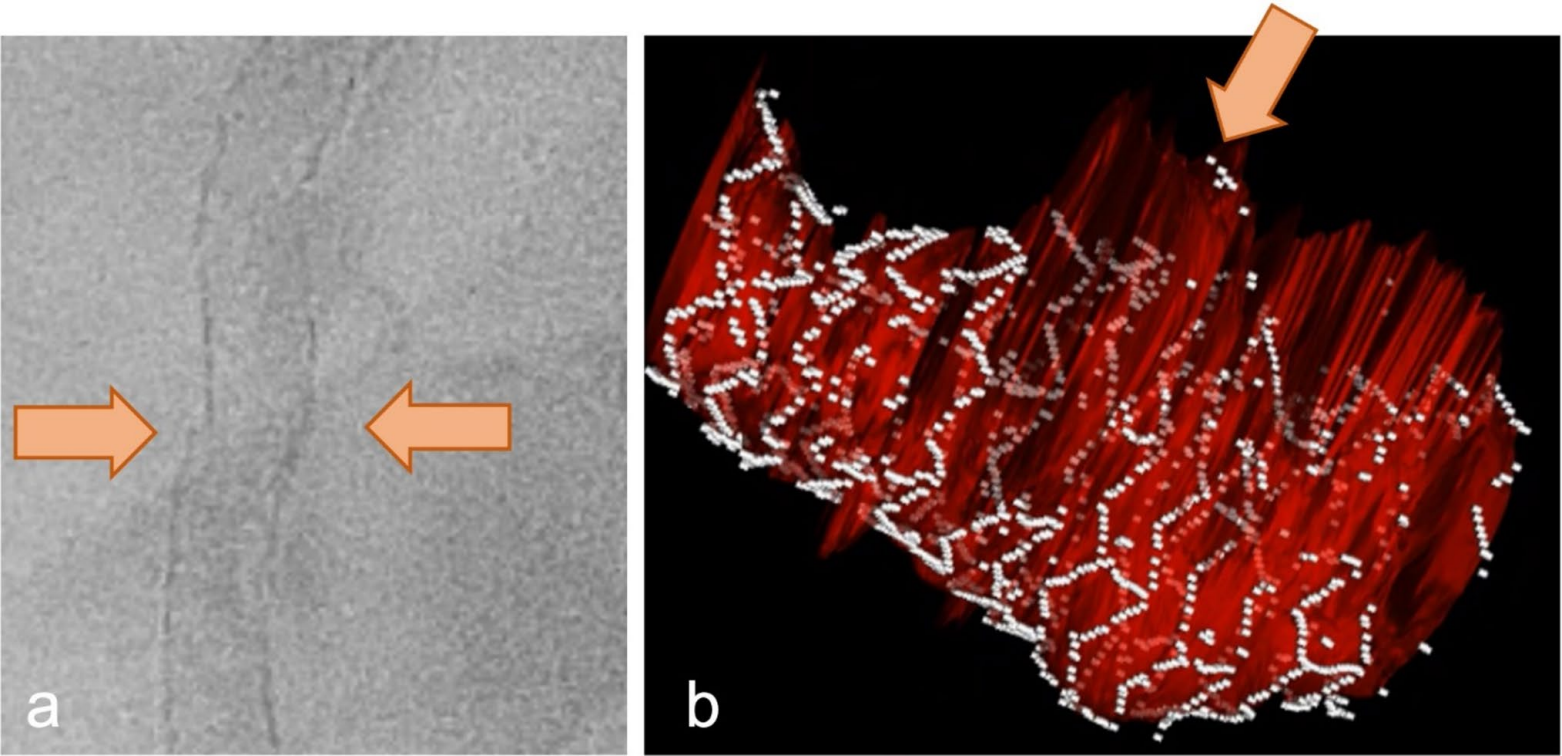
Angiography shows misalignment (*red arrows*) within a stent (**a**), confirmed at 3D reconstruction (**b)**

While tissue analysis might provide insight into the physiopathology of ISR, anatomical characteristics of stents provide the hemodynamic background for NIH and have also been associated with restenosis [103]. Mechanical issues involved in ISR may be corrected by imaging at the time of implantation (or diagnosed through imaging during follow-up, including stent under expansion. (Fig. 2) stent undersizing, geographic miss (i.e., failure to treat the complete longitudinal extent of disease) [104, 105], edge dissection (with the evidence being weaker for OCT than IVUS) [106], in-stent prolapse, malapposition (Fig. 3a) causing flow turbulence, inhomogeneous cross-sectional strut deployment (Fig. 3b) [107] and strut fracture. Of these parameters, the most convincing association with the perspective incidence of ISR is the incomplete deployment of a stent, resulting in a minimum stent area in the range of 4–5–5.5 mm^2^, as evidenced in the different studies [108, 109]. Of note, while these figures represent a minimum threshold, a linear association with a progressive decrease in the incidence of ISR is observed until a minimum stent area of about 8 mm2, beyond which a plateau is reached [110]. The evidence regarding the incidence, classification, and implications of strut fractures is less clear. Fracture in the metal strut modifies the device geometry, undermines its scaffolding properties, and it causes mechanical irritation (Fig. 4, 5)[111]. The resulting inflammation may elicit lipid deposition and be a possible cause of the development of neoatherosclerosis [112]. In line with this, stent fractures are reportedly associated with higher rates of ISR [51, 113]. In this scenario, high-resolution intravascular imaging coupled with 3D reconstruction can help identify fractures that are overlooked on angiography [114, 115]. Based on a pathological classification, our group recently proposed 4 different patterns of fracture, which were consistently associated with ISR or stent thrombosis at the fracture point [53]. Conclusively, calcifications, hinge points, and implantation pressures appear to be determinants of fractures.

## Treatment Strategies

**Fig. 6:**
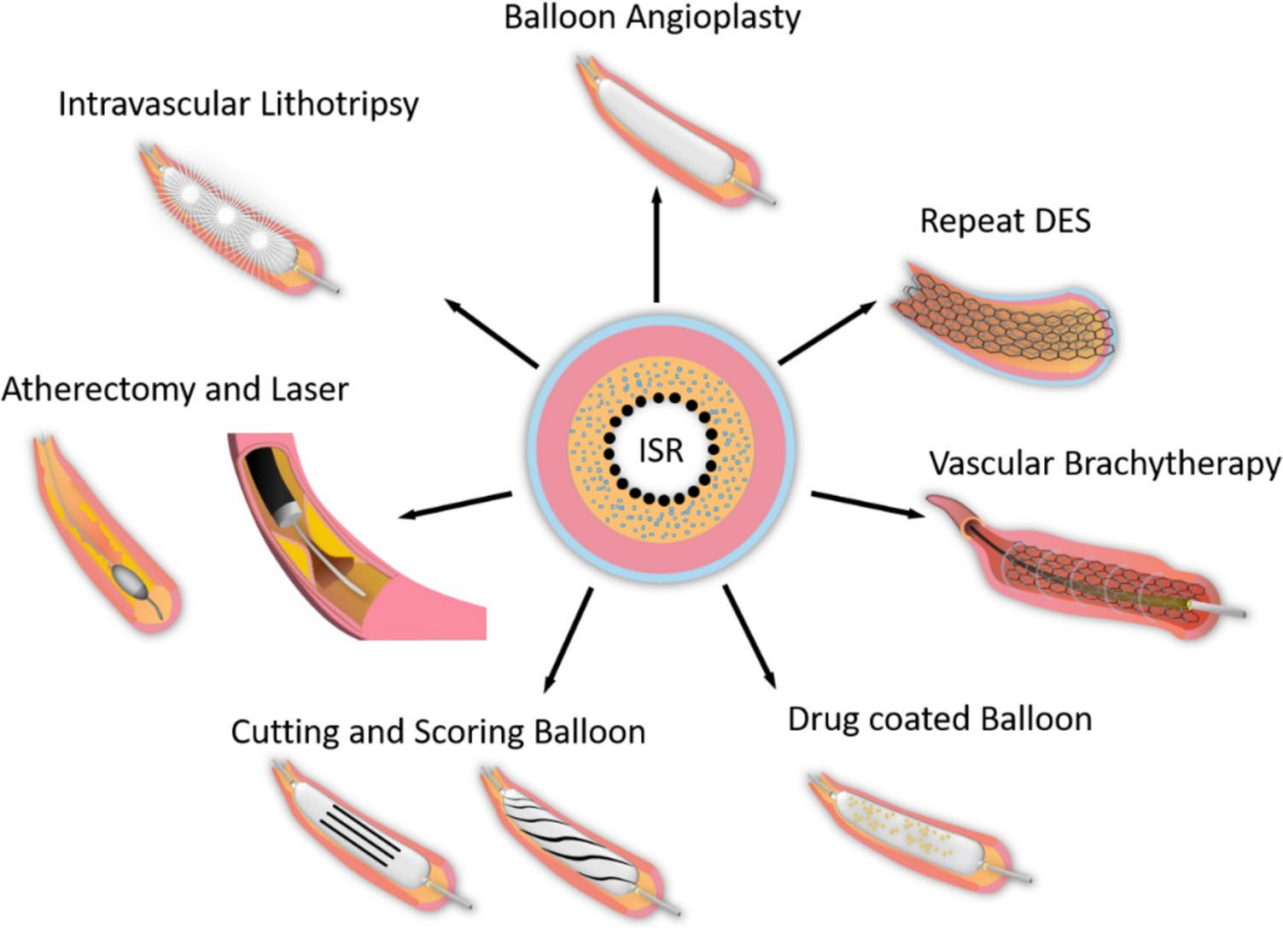
Treatment options for in-stent restenosis (ISR). DES— drug-eluting stent

ISR management is a critical challenge because a broad variety of mechanisms may be involved [1, 3]. Effective management of ISR requires a comprehensive approach that addresses both the underlying causes of ISR and the risk factors for recurrence (Fig. 6)[3]. Current treatment approaches for ISR have evolved significantly, emphasizing tailored therapies based on lesion type and underlying pathology.

## Balloon Angioplasty

Initially, plain balloon angioplasty was commonly used as the first intervention for ISR [1, 3]. However, this approach was associated with edge-related issues and a rate of ISR > 50% and it has almost been completely abandoned in Europe as a definitive therapy [116]. When dealing with a stent that has not been properly expanded, using non-compliant or ultra-high-pressure non-compliant balloons at high pressures (up to 40 atmospheres) is recommended to enhance stent expansion [117]. In a recent real-world data work, Kim et al. compared the long-term outcomes of drug-eluting balloon angioplasty and drug-eluting stent implantation in treating ISR [118]. They found no difference in target lesion revascularization between the two, but the drug-eluting balloon group had significantly lower rates of myocardial infarction and no target lesion thrombosis, suggesting a safer profile with similar efficacy [118]. However, the current evidence does not support using isolated balloon angioplasty (BA) as a standard treatment for ISR. Instead, this technique is best utilized for lesion preparation before implementing other therapies or for fine-tuning DES implantation [119].

## Cutting and Scoring Balloon

Cutting balloons are standard balloon catheters equipped with lateral metallic blades that, when inflated, incise the plaque in the stenotic area [120]. On the other hand, scoring balloons operate similarly but feature low-profile nitinol wires arranged in a spiral pattern on the surface of the balloon catheter [120]. Both cutting and scoring balloons may have a useful function in preparing the lesion before using DESs or DCBs to treat ISR. Studies have demonstrated that neointimal modification with a scoring balloon can enhance the anti-restenotic effectiveness of DCB therapy in patients with DES-ISR [121]. This is evidenced by reduced late lumen loss and similar clinical outcomes at 6 months in patients who underwent scoring balloon pre-dilatation compared to those receiving standard therapy [122]. On the other hand, cutting balloons have several advantages, including the theoretical ability to cut neointimal tissue using numerous blades to secure the balloon in the lesion and prevent the watermelon seeding effect [1, 3].

## Ablative Therapy

### Atherectomy and Laser

Rotational atherectomy (RA) is another ablative technique used to reduce ISR lesion size and facilitate subsequent treatments, particularly in cases of calcified neoatherosclerosis, as part of a combined therapeutic strategy [123]. Currently, there is a lack of randomized data regarding the use of RA in treating DES-ISR. Nonetheless, it can still serve as a complementary method for preparing the lesion before applying DCBs or carrying out recurrent DES implantation [124]. However, caution should be exercised in case of excentric neointima, such that metallic struts are exposed or close to the lumen, while successfully attempted in the literature, might represent an hazard for rotablation due to the risk of burr entrapment [125]. As an alternative to the use of larger burrs, "rotatripsy", ie the combination of rotablation with a small (< 0.5) burr to stent ratio followed by IVL (balloon to artery ratio closer to 1) has demonstrated to be safe in small case series [126]. Excimer laser coronary atherectomy (ELCA) employs monochromatic light radiation that generates shock waves and heat to disrupt and modify plaque. Data supporting the systematic use of ELCA as the primary treatment for ISR are limited, and there are no randomized studies on its effectiveness in treating DES-ISR [127]. However, in selected cases, it may serve as a complementary technique for lesion preparation, especially for recurrent ISR in cases with severe calcification. ELCA may facilitate stent expansion for patients with undilatable ISR due to heavily calcified arterial walls, particularly when contrast is injected to induce further barotrauma and microcavitation [128, 129]. Though evidence is derived from small observational series, ELCA remains a useful option as a last resort when other therapeutic strategies have been unsuccessful [130].

## Drug coated Balloons

Drug-coated balloons (DCBs) offer an anti-proliferative treatment option that does not necessitate the use of another metallic platform, but instead avoids multiple stent layers, thus making it an attractive solution for managing ISR [131–133]. DCBs may be especially beneficial in clinical scenarios where adding another stent layer is not ideal (such as multiple previous stent layers or the presence of a major side branch) and may be well-suited for treating ISR caused by stent malapposition [134]. DCBs are not available commercially in the United States for use in coronary arteries, but they are easily accessible elsewhere in the world. According to the European Society of Cardiology guidelines [135], DCBs are recommended as a therapy option for ISR because they reduce the need for an additional metallic layer (Class IA). DCBs include lipophilic medications, such as paclitaxel, which prevent neointimal hyperplasia. A recent meta-analysis found that DCB therapy is more effective in managing late DES-ISR, with significant improvements in major cardiac adverse events (MACE) and target lesion revascularization (TLR) outcomes at 12 months post-treatment [136]. While preliminary results from small exploratory clinical studies using sirolimus-based DCBs are promising [137], randomized trials are required to establish the efficacy of DCB treatment for DES-ISR [138]. Furthermore, Liu et al. conducted a prospective, multicenter, randomized, noninferiority trial to compare the clinical efficacy and safety of two different drug-coated balloons (DCBs)—Dissolve DCB and SeQuent Please DCB—in treating DES ISR [139]. Results demonstrated that the Dissolve DCB was noninferior to the SeQuent Please DCB concerning in-segment late loss. However, the study also noted numerically higher rates of target lesion failure and binary restenosis in the Dissolve DCB group compared to the SeQuent Please DCB group at 9 months, though these differences were not statistically significant [139]. In another recent non-inferiority trial by Chen et al., a novel biolimus-coated balloon (BCB) demonstrated similar efficacy to the paclitaxel-coated balloon (PCB) in treating coronary in-stent restenosis (DES-ISR), with comparable late lumen loss at 9 months. Clinical outcomes at 12 months were also similar between both treatment groups [140]. Eventually in a recent meta-analysis by Sedhom et al., comparing limus drug-coated balloons (DCBs) and paclitaxel DCBs for PCI, no significant differences were found in clinical outcomes such as target lesion revascularization [141]. However, paclitaxel DCBs demonstrated superior late angiographic outcomes, including lower rates of binary restenosis and late lumen loss [141].

## Repeated DES implantation

Several meta-analyses showed that using an additional DES for DES-ISR therapy resulted in better outcomes compared to angioplasty alone [89, 91]. In particular, everolimus-eluting stent (EES) implantation was the most effective treatment in improving diameter percentage stenosis at angiographic follow-up, followed by DCB [91]. Moreover, another meta-analysis showed that these second generation stents produced an almost significant 65% reduction in the risk of target lesion revascularisation compared with DCB [89]. The ISAR DESIRE-2 trial, which was the only randomized study conducted on this topic, did not demonstrate any advantage to using a different antiproliferative agent for treating sirolimus-eluting stent ISR [142]. On the other hand, the non-randomized RIBS-III study had indicated that the use of a different DES could result in better outcomes at 9-months follow-up [143]. Eventually, a recent meta-analysis found that PCI with DES for ISR carries a higher risk of adverse cardiac events compared to PCI with DES for de novo lesions [144].

## Vascular Brachytherapy

Intravascular brachytherapy (IVBT) provides localized radiation to prevent neointimal development within the stent [145–147]. While there has been a recent resurgence in the use of VBT for DES-ISR treatment, only a small number of institutions offer it. There is currently a lack of randomized data on the effectiveness of IVBT for treating DES-ISR. Some studies have shown promising outcomes with IVBT, but there is limited data on its effectiveness [148].

## Intravascular Lithotripsy

Intravascular lithotripsy (IVL) is a novel technology that employs localized pulsatile sound waves to modify vascular calcium in a circumferential manner [149]. IVL has shown to be both safe and effective in the treatment of de novo coronary artery lesions [150]. Although the use of IVL for stent expansion in ISR has been reported, there is limited data available on this technique, and its use is considered off-label. However, several observational studies have suggested that IVL may be successful in patients with undilatable ISR who are unresponsive to conventional therapies, particularly in cases of stent underexpansion due to circumferential coronary artery calcification [151]. Further studies are needed to evaluate the safety and efficacy of IVL in the treatment of ISR and to determine its optimal role in the overall management of this challenging condition.

## Guidelines

Current European Guidelines for chronic coronary syndromes highlight that for ISR in BMS, DCB angioplasty and repeat DES implantation have shown comparable efficacy and safety [152]. However, in the context of DES ISR, paclitaxel-eluting stents demonstrated superior outcomes over DCB angioplasty, particularly in reducing the risk of restenosis in the short term [152, 153]. At 10 years, however, studies indicated no significant differences in major clinical endpoints between DCB angioplasty and repeat DES implantation [154]. Both approaches consistently outperformed plain balloon angioplasty in reducing target lesion revascularization, a critical marker of procedural success [152]. Among DES options, everolimus-eluting stents have emerged as the frontrunner, with evidence showing better long-term outcomes compared to DCBs [152]. At the end, European guidelines recommend DES over DCB for DES ISR (Class 1A) [152].The American College of Cardiology (ACC)/American Heart Association (AHA) guidelines recommend repeated DES as a Class IA indication for the treatment of ISR [155]. However, it is important to note that DCBs are not approved for commercial use in coronary artery interventions in the United States, which limits their availability for the treatment of ISR. Interestingly, both the European and U.S. guidelines also recommend the use of intracoronary imaging with IVUS or OCT as a Class IIa indication to help elucidate the mechanisms of ISR [135, 155]. This can help guide the selection of the most appropriate treatment strategy and improve outcomes for patients.

## Antiplatelet Therapy

Regarding antiplatelet therapy, in the field of ISR it can vary depending on the treatment approach: for instance a secondary analysis from the PRODIGY trial showed that 24 months of DAPT reduced death, myocardial infarction, and stroke rates compared to 6 months, in patients treated with DES implantation, whereas for those treated with DCB, major RCTs suggest DAPT durations of 3 to 12 months [156, 157]. A shorter DAPT may be considered for those treated with DCBs or cutting BAs, especially if they are at a higher bleeding risk [3].

## Prediction Tools

Coughlan et al. have recently developed and validated the ISAR score, a predictive model designed to estimate the risk of undergoing repeat PCI for recurrent DES-ISR [158]. The analysis included 1,986 patients, identifying four critical factors associated with the necessity for repeat PCI within a year: non-focal ISR pattern, early ISR occurrence (within six months), ISR affecting the left circumflex artery, and ISR in calcified vessels [158]. The ISAR score's predictive accuracy was shown to be superior to that of existing ISR classification models, making it a valuable tool for predicting the 1-year risk of repeat PCI due to recurrent DES-ISR [158]. Furthermore a recent study explored the possible use of machine learning to predict in-stent restenosis ISR risk in patients with ACS undergoing PCI [159]. By analyzing a cohort of 340 subjects, the study found that significant predictors of ISR included the number of affected arteries (two or more), stent generation, and stent diameter [159].

## Proposed Algorithm of ISR Treatment

**Fig. 7:**
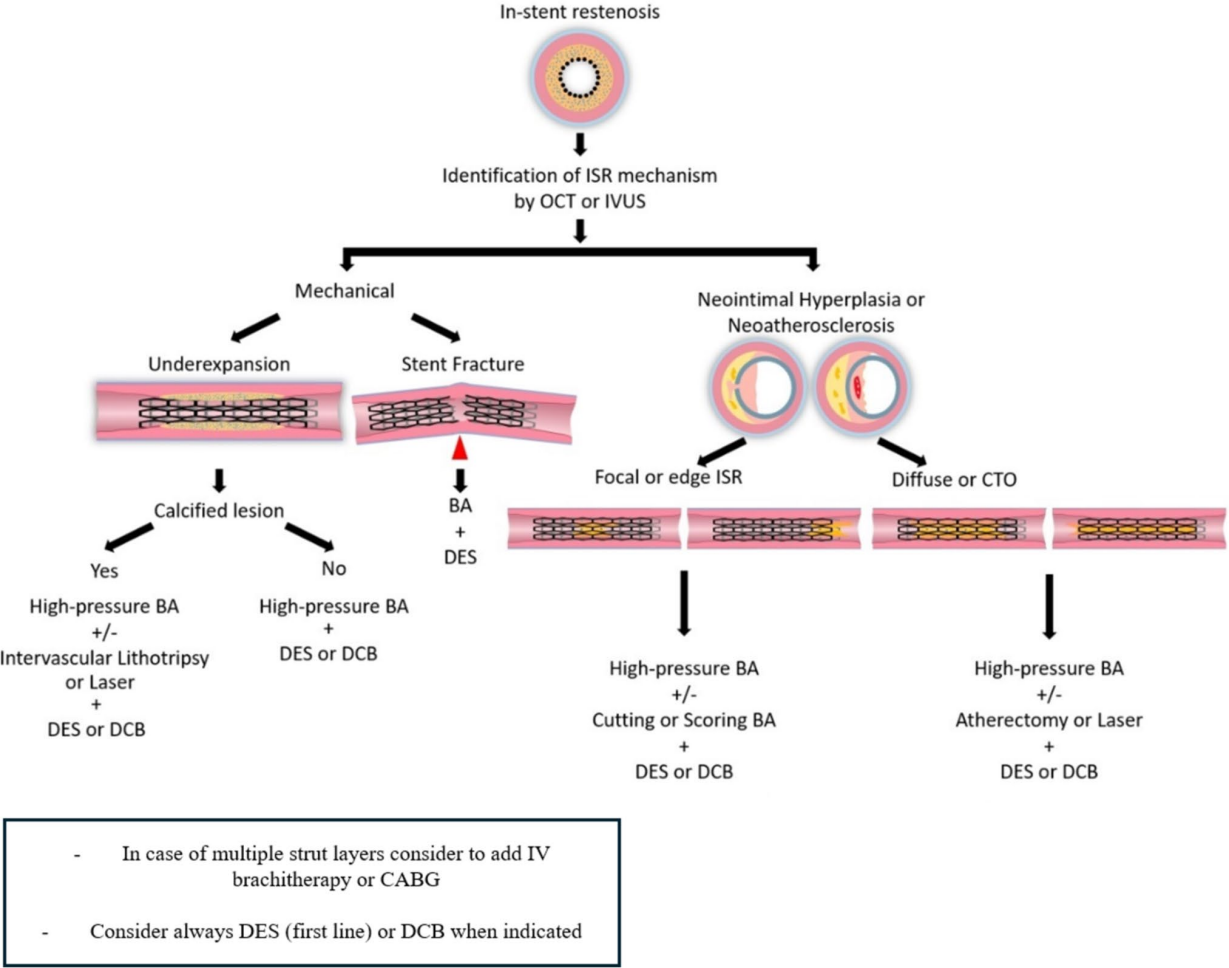
Proposed algorithm of treatment

Here we propose a therapeutic algorithm for the management of ISR based on current evidence and guided by findings from intracoronary imaging modalities, such as OCT and IVUS (Fig. 7). For cases of ISR caused by mechanical issues, different strategies are recommended depending on the specific pathology. If ISR is due to stent underexpansion, particularly in the presence of a calcified lesion, treatment should begin with high-pressure balloon angioplasty. Adjunctive therapies, such as intravascular lithotripsy or laser, may be necessary to address resistance from calcific deposits. Once sufficient expansion is achieved, either a DES (first line) or a DCB can be applied to reduce the likelihood of recurrence. In cases where ISR is associated with stent fracture, high-pressure BA is again recommended, followed by the placement of a DES or DCB to stabilize the affected segment and restore vessel patency. When ISR arises from biological causes, such as neointimal hyperplasia or neoatherosclerosis, the treatment approach varies based on lesion morphology. For focal or edge ISR due to these biological processes, high-pressure BA with the addition of a cutting or scoring balloon is beneficial to modify the neointimal tissue. After lesion modification, either a DES or DCB can be deployed to minimize restenosis risk. However, in diffuse ISR or cases of chronic total occlusion (CTO), where neoatherosclerosis often plays a role, more aggressive lesion preparation may be required. High-pressure BA combined with atherectomy or laser therapy is particularly valuable for treating calcified neoatherosclerotic plaques, and, following adequate lesion preparation, a DES or DCB should be used to promote long-term patency. For complex ISR cases involving multiple stent layers or particularly resistant ISR patterns, additional interventions, such as intravascular brachytherapy (IVBT) or coronary artery bypass grafting (CABG), may be considered.

## Conclusions

ISR continues to pose a significant clinical challenge with DESs and remains difficult to treat effectively. The most promising approach to minimizing ISR may lie in using imaging-guided techniques to optimize stent placement during deployment. Intracoronary imaging plays a crucial role in identifying the underlying mechanism of ISR, allowing for tailored therapy. Given the diverse conditions associated with ISR, no single treatment strategy is universally effective.

## Key References

- Shlofmitz E, Case BC, Chen Y, et al. Waksman In-Stent Restenosis Classification: A Mechanism-Based Approach to the Treatment of Restenosis. Cardiovasc Revasc Med 2021;33:62-67. 10.1016/j.carrev.2021.06.004.

- **Findings from this study highlight the importance of the mechanisms underlying ISR for its treatment.**

- Giustino G, Colombo A, Camaj A, et al. Coronary In-Stent Restenosis: JACC State-of-the-Art Review. J Am Coll Cardiol 2022;80(4):348–372. 10.1016/j.jacc.2022.05.017.

- **This paper is a comprehensive review of ISR.**

## Data Availability

No datasets were generated or analysed during the current study.
